# Estimating additional health and social costs in eating disorder care for young people during the COVID-19 pandemic: implications for surveillance and system transformation

**DOI:** 10.1186/s40337-024-01003-1

**Published:** 2024-04-26

**Authors:** Nicole Obeid, Jennifer S. Coelho, Linda Booij, Gina Dimitropoulos, Patricia Silva-Roy, Mary Bartram, Fiona Clement, Claire de Oliveira, Debra K. Katzman

**Affiliations:** 1 Eating Disorders Research Lab, Children’s Hospital of Eastern Ontario Research Institute, 401 Smyth Rd, K1H 8L1 Ottawa, ON Canada; 2 Department of Psychiatry, University of Ottawa, Ottawa, ON Canada; 3 Provincial Specialized Eating Disorders Program for Children & Adolescents, BC Children’s Hospital, Vancouver, BC Canada; 4 Department of Psychiatry, University of British Columbia, Vancouver, BC Canada; 5 Eating Disorders Continuum, Douglas Mental Health University Institute, Montreal, QC Canada; 6 Department of Psychiatry, McGill University, Montreal, QC Canada; 7 Calgary Eating Disorder Program, Alberta Health Services, Calgary, AB Canada; 8 Department of Psychiatry, University of Calgary, Calgary, AB Canada; 9 Mental Health Commission of Canada, Ottawa, ON Canada; 10 School of Public Policy Administration, Carleton University, Ottawa, ON Canada; 11 Department of Community Health Sciences, O’Brien Institute for Public Health, University of Calgary, Calgary, AB Canada; 12 Institute for Mental Health Policy Research, Centre for Addiction and Mental Health, Toronto, ON Canada; 13 Institute of Health Policy, Management and Evaluation, Dalla Lana School of Public Health, University of Toronto, Toronto, ON Canada; 14 Division of Adolescent Medicine, Department of Pediatrics, Hospital for Sick Children, Toronto, ON Canada; 15 Department of Pediatrics, University of Toronto, Toronto, ON Canada

**Keywords:** Eating disorders, COVID-19 pandemic, Costing study, Young people, Health costs, Social costs, Support-based community eating disorder organizations, Surveillance

## Abstract

**Background:**

The impact of the COVID-19 pandemic on young people with eating disorders (EDs) and their families was profound, with surging rates of hospitalizations and referrals reported internationally. This paper provides an account of the additional health and social costs of ED care for young people living in Canada incurred during the COVID-19 pandemic, drawing attention to the available data to inform these estimates while noting gaps in data capacities to account for a full view of the ED system of care.

**Methods:**

Three methodologies were used to capture costs: (1) provincial administrative data holdings available at the Canadian Institute of Health Information (CIHI) were used by Deloitte Access Economics to conduct analyses on costs related to hospitalizations, emergency room visits, outpatient visits with physicians and loss of well-being from being on a waitlist. These were examined across three fiscal years (April 1 to March 31, 2019–2022) to compare costs from one year before to two years after the onset of the pandemic, (2) data collected on support-based community ED organizations and, (3) costs identified by young people, caregivers and health care professionals.

**Results:**

Estimates of additional health care costs and social costs arising from ED care waitlists were estimated to have increased by 21% across the two years after the onset of the pandemic and is likely to represent an underestimate of costs. Costs related to some standard ED care services (e.g. day treatment programs) and support-based community ED organizations that saw a 118% increase in services during this time, are some examples of costs not captured in the current cost estimate.

**Conclusions:**

This paper provides a first account of the additional health and social ED care costs associated with the pandemic, which indicate at minimum, a 21% increase. The results invite discussion for more investments in ED services for young people in Canada, as it is unclear if needs are expected to remain elevated. We suggest a call for a national surveillance strategy to improve data holdings to aid in managing services and informing policy. A robust strategy could open the door for much-needed, data-informed, system transformation efforts that can improve ED care for youth, families and clinicians.

**Supplementary Information:**

The online version contains supplementary material available at 10.1186/s40337-024-01003-1.

## Abstract (fr)

The impact of the COVID-19 pandemic on young people with eating disorders and their families was profound, incurring substantial costs to youth, families, health professionals, eating disorder systems of care, and society. This study looks to draw on available data in Canada to estimate the additional health and social costs experienced during the pandemic. Using available national data holdings on eating disorder health system use, survey and discussion groups with individuals who provided or received eating disorder care during the pandemic, and data from support-based community eating disorder organizations, we attempted to capture the fulsomeness of these costs and demonstrate gaps that are likely leading to an incomplete picture. Available data across Canada on hospitalizations, emergency room visits, outpatient visits and loss of wellbeing while on a waitlist were looked at across three fiscal years (April 2019 to March 2022). Overall, an average increase of 21% of additional health care and social costs was seen across the two years after the onset of the pandemic, although this is considered an underestimation. Additional costs such as those for other standard eating disorder treatment options (e.g. day treatment programs) were not captured in these estimates. Additionally, costs related to services received through support-based community eating disorder organizations that saw a 118% increase in services during the pandemic, and private services (e.g. psychologist, dietitian) are some further examples of costs not captured in the current cost estimate. The results of this study highlight that needs and costs related to ED care rose during the pandemic and provides the first account of by how much. Given data limitations, there is a need to revisit national surveillance strategies for eating disorders to help increase accuracy of these estimates and aid in managing services and informing policies related to eating disorder care.

## Introduction

The COVID-19 pandemic has had a severe impact on children and youth with eating disorders (ED) and their families, with studies from across the globe demonstrating a significant increase in the number and severity of new and pre-existing EDs compared to prior years. This crisis has resulted in higher rates of new cases [1], hospital admissions, and emergency room visits [2, 3] with youth in treatment reporting increased eating disordered thoughts and behaviours and decreased motivation for recovery [4]. Undoubtedly, the impact of the pandemic has unmasked a global ED public health crisis.

The changes seen during the pandemic revealed how unprepared the already under-resourced Canadian ED healthcare system was to handle the unprecedented surge in the volumes of young people with EDs [5, 6]. Waitlists for ED care were documented to be at a record high in Canada [5, 6] and internationally [7], providing a glimpse into the unmatched need during the pandemic. The absence of comprehensive national ED surveillance data restricted the ability to identify shifting trends in the prevalence, health service utilization and waitlists of individuals with EDs [8], resulting in limited real-time data available to plan effectively for changing service needs. These gaps in data also led to the inability to quantify costs related to these shifts, likely translating to missed opportunities for the reallocation of scarce resources, and for leveraging this data to policymakers in order to signal when EDs need to be made a priority.

Despite health service studies conducted during the pandemic consistently reporting massive shifts in healthcare needs for young people with EDs that coincide with the onset [7], there has not yet been an analysis of the increased health and social costs associated with this surge. Pre-pandemic studies on the prevalence and social and economic costs for EDs have been carried out in the United States (US) [9], the United Kingdom [10] and Australia [11] with estimates reported in the billions of dollars per year related to health system costs, individual and family productivity costs, lost well-being and societal economical costs (USD). With all three of these reports conducted pre-pandemic, we are limited in our understanding of the costs related to the increased care needs seen during the pandemic.

In Canada, specialized ED services are publicly funded and provincially managed, with disproportionate services available across provinces and territories [12]. Services typically include inpatient, residential, day treatment, and outpatient services, with often long waitlists [12]. Health system utilization data is collected at the provincial level and collated nationally by the Canadian Institute of Health Information (CIHI). National-level prevalence and economic studies on the costs of EDs prior to or during the pandemic in Canada have not been carried out, despite being identified as a priority by ED stakeholders in Canada [13]. Accordingly, over 40 ED experts and stakeholders (individuals and family members with lived experience, researchers, clinicians, decision-makers and leads of ED organizations) from across Canada came together to collaborate on a study of the additional health and social costs that were associated with the pandemic to help provide some general understanding of costs of EDs in Canada, how this shifted with the pandemic, and how this may help inform any future service disruption preparedness plans. The objectives of this larger project included gathering input from a range of sources and stakeholders on ED costs related to the pandemic by (1) commissioning Deloitte Access Economics, the same firm that conducted the Australian [11] and US [9] social and economic costing studies, to carry out an economic analysis of the *additional ED related* health and social costs using national administrative holdings from across Canada, (2) use surveys and follow-up discussion groups to gather reports of direct medical, indirect medical and indirect costs experienced by clinicians, decision-makers, and youth/families with EDs during the pandemic (detailed findings reported in a joint publication led by Obeid and colleagues [14]), and 3) collecting information about additional service use provided by support-based community ED organizations for young people with EDs living in Canada to help understand those costs not currently captured in administrative databases or other sources.

Our goals in this paper are to bring together these findings to highlight the different types of costs that were reported across stakeholders across these various methodologies in attempts to capture a fulsome view of additional costs experienced during the pandemic. We use this information to further highlight the data gaps and their impacts on costing estimates and service planning and delivery. Based on these findings, we share recommendations for a national surveillance strategy to address these gaps with goals to help strengthen the capture of more accurate data to inform future costing and prevalence studies, and to aid with planning and management of ED resources.

## Methods

### Summary of economic analysis of additional health and social costs

The analysis carried out by Deloitte Access Economics [15] (The impact of COVID-19 on eating disorders among Canadian youth: https://www.deloitte.com/au/en/services/economics/analysis/impact-covid19-eating-disorders-among-canadian-youths.html) was based on their modelling approach, similarly used in other costing studies [9, 11], using data holdings obtained from the Canadian Institute of Health Information (CIHI). Data was obtained from CIHI through a data request process identifying databases and characteristics of the required data (i.e. age, diagnostic code…etc.). Deloitte assessed costs (in 2023 CAD) associated with the shifts in healthcare utilization (hospitalizations, emergency room, and outpatient visits) and loss of wellbeing due to being on a waitlist across the pandemic. Given that the average age of diagnosis for EDs falls within adolescence and young adulthood [16] and that the pandemic disproportionately affected the rates of young people presenting with EDs [17, 18], we focused on EDs in young people aged 5–25 years. The Deloitte analysis examined national administrative ED data across three fiscal years (2019–2022). It included data on individuals with an ED diagnosis (listed within the first three diagnoses recorded) of anorexia nervosa (F50.0, F50.01, F50.02); atypical anorexia nervosa (F50.1); bulimia nervosa (F50.2, F50.3); binge-eating disorder (F50.81); avoidant/restrictive food intake disorder (F50.82) or other or unspecified eating disorders (F50.8, F50.9, F50.89). The following databases were used: the Discharge Abstract Database (DAD), the National Ambulatory Care Reporting System (NACRS), the Hospital Morbidity Database (HMDB), and the Ontario Mental Health Reporting System (OMHRS). Available data on hospitalizations, emergency room, and outpatient visits were included. Extracts were broken down quarterly across the three fiscal years (April 1-March 31 2019/20, 2020/21 and 2021/22), which covered approximately one year before the pandemic (April 1, 2019, to March 31, 2020) and two years after the onset of the pandemic (April 1, 2020, to March 31, 2022).

Additional costs relating to individuals’ loss of wellbeing due to wait times were also estimated (see Appendix for more details and/or page 14 of the full Deloitte report) [15]. Wait times were derived from the Fraser Institute’s [19] estimate of time from referral to entry into specialized ED programs. Loss of well-being due to waiting for care was calculated by multiplying (1) the total number of people waiting for treatment as per the CIHI inpatient waitlist, by (2) the average number of days spent waiting (Fraser report), by (3) the daily loss of wellbeing (based on disability adjusted life years (DALYs) that relied on estimates for EDs obtained from the Institute for Health Metrics and Evaluation (IHME) Global Burden of Disease Study 2019 [20], by (4) the value of an additional day of perfect health (based on value of a statistical life year informed using the Canadian Cost-Benefit Analysis guidelines [21].

### Survey and discussion groups

As reported in an adjoining manuscript [14], reports of costs collected via conducting surveys (*n* = 117) and follow-up discussion groups (*n*= 21) with youth with lived experience, caregivers, clinicians, and decision-makers was also conducted. While details on these findings are shared in the separate publication, incorporation of these costs at a high level were included here to allow for a thorough integrated investigation of the larger cost context during the pandemic in Canada.

### Data capture from support-based community ED organizations

Service delivery data from support-based community ED organizations (e.g., text/chat crisis support lines, peer support groups, etc.) in Canada were requested to articulate some of the shifts seen in these settings across the pandemic. Support-based community ED organizations from across Canada were invited to share data via survey across three fiscal years to align with the years examined in the Deloitte report (April 2019 to March 2022). The survey was circulated from December 2022 to February 2023 regarding number of new hires (staff), number of patients seen across all programming, dollars spent on training, number of requests made for education sessions, number of referrals to other organizations, number of calls/online chat requests, and website traffic. Data was received from eight organizations (53% of those asked; see Table 1), three of whom identify as national organizations and five who operate provincially/regionally.

## Results

### Main findings from deloitte analysis of health and social costs

The Deloitte report indicated that nationally, hospitalizations increased by 60% (from 1,514 to 2,416 visits) from the year before the pandemic (April 1, 2019 to March 31, 2020) to the average of the two years after the onset of the pandemic(April 1, 2020 to March 31, 2022). Estimates extrapolated based on data from two provinces (Ontario and Alberta, representing approximately 50% of the population in Canada [22]) also demonstrated a marked increase in emergency room visits with the onset of the pandemic, with presentations rising to an average increase of 126% (from 1,019 to 2,301 visits) in the two years following the pandemic (April 1, 2020 to March 31, 2022) compared to the year before the onset (April 2019 to March 2020). Data on medical outpatient visits, extrapolated from data from Alberta only, showed a 3% decrease (from 148,252 to 144,283 visits) of in-person outpatient visits across the first two years of the pandemic, which contrasts with the exponential (11,432%) increase seen in virtual visits two years after the onset of the pandemic. Together, hospitalization, emergency room, and outpatient visit costs resulted in an additional $20.2 million dollars in costs to the ED system of care. This is an 18.3% increase over the first two years of the COVID-19 pandemic, with care for females 5 to 17 years representing the majority of these costs (68% of costs in the year before the pandemic, increasing to 76.5% and in the two years after the onset of the pandemic).

Wait times to see an ED specialist was calculated to have increased by 6 weeks across the two years after the start of the pandemic. This led to an *additional* cost of $2.6 million and $16.7 million in the first and second year of the pandemic, respectively, totaling an additional $19.3 million of wait time costs over the two years.

Thus, the total *additional* costs of hospitalizations, emergency room visits, medical outpatient visits and wait time costs were estimated at $39.5 million over two years after the onset of the pandemic (adjusted for inflation). This represents an *additional* 21% increase in costs associated with the shifts in ED care seen during the first two years after the start of the pandemic (total cost FY 2019/2020=$96.1 million compared to total cost in FY 2021/2022=$130.8 million).

### Survey and discussion groups

Across surveys, the pandemic related costs that were identified by respondents as either new types of costs incurred as a direct result of the pandemic or as pre-existing costs that were exacerbated by the pandemic included (1) increased costs for accessing private services given cancelled, delayed or inaccessible ED care (psychologists, dietitians, etc.), (2) medication costs, (3) costs related to needing to purchase special food or supplements, (4) transportation costs, (5) costs related to not being able to attend school/work, (6) costs related to the increased isolation associated with the restrictions of the pandemic (e.g. lack of support from extended family members), and (7) costs related to inability to find an ED practitioner during these crisis times. Findings from the discussion groups further revealed costs related to (1) decreased accessibility of services, (2) costs to care for other family members/siblings affected, (3) costs related to cancelled or delayed treatments due to the pandemic, and (4) prohibitive treatment costs being the only option. Healthcare professionals additionally reported extensive challenges with resources and increased work expectations due to the pandemic context that led to costs. They shared increasing responsibilities as it relates to both shifting and providing virtual services as well as responding to the increasing demand for services contributed to personal costs.

### Data capture from support-based community ed organizations

Data provided by Canadian organizations demonstrate estimates of the shift in care that occurred with the pandemic in these community-based settings; data not captured by current administrative holdings. Table 1 provides service utilization data from support-based community ED organizations across the fiscal years studied. On average there was an 88% increase in the number of hits/clicks on websites, an increase of 100% in number of calls/online chat requests, and an average of a 118% increase in individuals seen across all programming when comparing data from the year before the pandemic (April 1, 2019, to March 31, 2020) to two years after the start of the pandemic (April 1, 2020 to March 31, 2022).

**Table 1:** Percent increase from FY1 (April 1, 2019, to March 31, 2020) to FY2 (April 1, 2020 to March 31, 2021) and FY1 to FY3 (April 1, 2021 to March 31, 2022) among support-based community ED organizations in Canada

	Description of organization	Increase of hits/clicks on website			Increase of calls or online chat requests			Increase of individuals across programs	
		FY1-FY2	FY1-FY3		FY1-FY2	FY1-FY3		FY1-FY2	FY1-FY3
National Eating Disorder Information Centre	Provides information, resources, referrals and support to anyone in Canada affected by an ED [23].	-1%	26%		56%	108%		-2%	37%
National Initiative for Eating Disorders	Develops and shares educational resources and information, contributes to research, and takes action to address the needs of Canadians impacted by EDs [24].	108%	133%		100%^1^	121%^1^		N/A	N/A
Body Brave	A charity providing accessible ED treatment and support, as well as advancing community training and education [25].	125%^2^	233%^2^		56%	277%		32%	283%
BridgePoint Center for Eating Disorder Recovery	A provincial resource based out of Saskatchewan providing recovery-based programming for people who are experiencing disordered eating [26].	11%	3		-11%^3^	42%^3^		-2%^4^	108%^4,5^
Eating Disorders Nova Scotia	A community based, charitable organization that believes everyone should have access to the supports they need for recovery. All of their services are available without a referral or a diagnosis [27].	N/A	N/A		N/A	N/A		55%	131%
Eating Disorder Support Network of Alberta	EDSNA offers professionally facilitated support groups to those (18+) affected by EDs /disordered eating, and their caregivers [28].	29%	77%		N/A	N/A		198%	214%
Kelty Mental Health Resource Centre	Provides information, resources and peer support to people across British Columbia with an ED or disordered eating concern [29].	12%^6^	9%^6^		N/A	N/A		87%	93%
Silver Linings	Silver Linings Foundation works collaboratively to bridge gaps in accessibility and care of EDs in Alberta [30].	22%	289%		30%	73%		80%	212%
Average change per year		**49%**	**127%**		**47%**	**153%**		**75%**	**162%**
Average change across years (FY1 to FY2 + FY3)		**88%**			**100%**			**118%**	

FY = financial year

^1^number of people reached through online community (e.g., Google, website, social media outlets)

^2^number of program emails

^3^does not include the number of discrete individuals supported by phone or email

^4^FY3 includes residential and virtual care programs

^5^unique page views

^6^number of users on website

## Discussion

This study represents the first attempt to quantify the additional health and social costs related to the shifts in ED care seen during the COVID-19 pandemic. The Deloitte report estimated an additional 21% increase in health and social costs due to EDs in Canada during the first 2 years of the pandemic, quantifying for the first time the additional cost of the pandemic as it relates to ED care. While this is a sizeable additional cost, we ascertain that this increase is only a partial view of the overall costs given noted data gaps, limiting our understanding of the true impact of the pandemic on these costs. Further, a number of other highly impactful, uncaptured costs were identified by those receiving or providing ED care in Canada, with some consisting of costs specific to the COVID context (e.g. closed or delayed programming) versus other costs representing pre-existing ones that were likely magnified or exacerbated during the pandemic (e.g. longer waitlists). Costs absorbed by vital support-based community organizations that in some areas saw a doubling in usage, also supports these figures representing a partial view. This initial attempt to quantify the additional social and economic ED costs related to the pandemic provides a starting point in which to launch discussions about the shift in ED costs seen in the context of the pandemic, providing signals to help with future preparedness planning. It also springboards discussions in Canada of available data to inform costing studies and stimulates conversation of how we can continue to find optimal ways to quantify these costs.

Some contributors to the underestimation in these cost amounts relates to the limitations identified by Deloitte when conducting their analysis (see Appendix for more details of identified limitations). The report acknowledges data suppression, data omissions, limits with the use of the Fraser Institute report to estimate wait times given its non-representativeness, and no available data on some types of EDs as potentially contributing to underestimation. Similarly, data from support-based ED organizations and identification from those with lived experience, caregivers and clinicians of other important costs that arose in the context of the pandemic [14], points to the need to systematically capture and understand how much is being spent on other ED services not currently captured in surveillance efforts, to better understand the full complement of costs and needs around ED care. The ability to systematically capture many more of these costs in Canada would contribute significantly to service, policy and surveillance efforts. It would similarly provide the opportunity to identify inequities in access to appropriate services across Canada [31], providing further important data to inform service decisions and policy.

Other sources of underestimation are related to the challenges in capturing all individuals with EDs within national administrative datasets, given the recognized constraints with the current diagnostic coding system. For instance, several of the national administrative datasets (i.e., DAD, NACRS and HMDB) rely on the ICD-10-CA codes, which do not have specific coding for avoidant/restrictive food intake disorder or binge-eating disorder. As a result, it is not currently possible to capture system-level data related to these important diagnostic groups. Additionally, individuals who present for medical care sometimes have a medical diagnosis (e.g., bradycardia) recorded as the reason for presentation in a health care setting and therefore EDs are not listed as the main diagnoses. A recent Canadian study [8] showed how clinically relevant cohort numbers varied in administrative datasets when using different search strategies for the identification of individuals with an ED. The researchers ran three different search strategies and reported that the number of cases more than doubled (from 7,268 to 17,313) when using ED diagnosis as primary concern only versus when identifying using ED diagnosis as any diagnosis and/or had an emergency room visit with an ED diagnosis. Prioritizing the standardization of how best to identify ED cases in health administrative data holdings and the endorsement for use of the most recent set of diagnostic codes across all provinces and datasets can remedy some of these identification issues.

A further gap in our national data holdings is the ability to capture the usage and associated costs of ED-related care across the full-service continuum. While not unique to the field of EDs, national CIHI administrative data capture is overwhelmingly tied to hospitalizations or emergency room visits, limiting the capture of ED care of publicly-funded outpatient hospital-based visits with allied health professionals offered in-person or virtually (e.g., psychologists, social workers, dietitians, and occupational therapists), intensive outpatient and day treatment programs, and residential care; programming that is considered standard to ED care in Canada [12]. Similarly, CIHI data holdings are equally not able to capture ED care in the community. This includes visits in primary care offices, recognized as the highest contributor to ED health system costs in several studies [32] including the US ED costing report [33] ($3.4 billion USD versus $0.8 million for residential care and $0.24 billion for emergency department visits and hospitalizations). This also includes services received from support-based community ED organizations. How much these omissions contribute to the partial view of estimations in the Canadian context remains unknown. Mechanisms to capture ED service provision in primary care and other support-based community settings should be prioritized. Collaborating and co-designing these efforts with interested parties in these settings to create consensus about goals, types of measures and use of such data would likely facilitate implementation and adherence.

Comparing to other social and economic studies conducted internationally (US, UK and Australia), the Canadian report focused on the *additional* costs incurred with the pandemic and as such included only available data on hospital-based service utilization costs and loss of well-being costs from being on a waitlist that shifted with the onset of the pandemic. Other reports, such as the Australian costing reports (2012; 2024) [11, 34] had a number of other costs included that provided a fuller account of costs including healthcare usage, loss of productivity (reduced employment, absenteeism, presenteeism, premature mortality, search and hiring, informal care), efficiency losses, other costs such as groceries, and expanded definitions of loss of well-being that included premature mortality and quality of life. In all reports, loss of well-being and loss of productivity costs were by far the highest contributors to costs, with the most recent Australian report estimating loss of well-being as representing 69% of costs and loss of productivity representing another 27%. This suggests that further costing studies in Canada should aim to expand to account for these costs which would likely increase the costs estimates substantially. Additionally noted is the fulsomeness of data in the Australian report for example, which was able to capture more complete health system data via the Australia Institute of Health Welfare. This included costs from state and territory funded services, private health insurance costs, out-of-pocket expenses (e.g. groceries), private and public hospital services, primary health care visits and specialists visits, and prescription pharmaceuticals. A surveillance strategy in Canada that would allow for the systematic capturing of these healthcare costs would enhance our ability to more accurately account for costs. Accounting further for the large percentage (estimated at 76.8% of those with diagnosable ED) of young people who never seek ED care [35] and individuals over the age of 25 years who are not included in this report would likely also increase these figures.

Valuable lessons were gleaned from conducting this study in terms of our understanding of the cost impacts related to the pandemic for ED care in Canada and our ability to carry out a costing study on ED care in Canada. This study highlighted important components that are essential for accurate costing estimates using CIHI data across periods of normal service delivery or times of crisis, encouraging standardization of these components for more comprehensive surveillance of the ED system of care. Essential to this are (1) a diagnostic classification system adopted by all provinces and territories that can be used to easily identify the various diagnostic categories of EDs across all data capture systems, (2) a national health administrative database capture that is comprehensive and inclusive of all aspects of ED care including publicly funded support-based community providers (e.g., National Eating Disorder Information Centre), multidisciplinary health providers in hospital- or community-based ED outpatient settings (e.g. dietitians), primary care practitioners, intensive outpatient and day treatment programs, and residential care, and (3) strategies to encourage mandatory reporting standards from all jurisdictions so that policy and decision-makers are able to have a cross-country understanding of prevalence and costs and where resources are being directed. Together, these mechanisms can encourage a minimum data set reporting that would enable important estimation and national surveillance for the field that would be paramount for any system transformation efforts.

## Conclusions

The pandemic had a significant effect on young people with EDs and their families in Canada. This put enormous pressure on the ED health care system and individuals, highlighting opportunities for better organization of ED care in Canada during times of crisis. The shift in service care needs resulted in an additional 21% increase in health and social costs over the two years after the onset of the pandemic. We further observed service use in support-based community ED organizations more than double, and additional costs related to accessing private services given cancelled, delayed or inaccessible programming, as further significant impacts during the pandemic. While the cost estimates presented only a partial view of the true health and social costs of ED care during the pandemic, the estimates from the report [15] together with the complementary data from individuals with lived experiences and support-based community organizations, provided the first systematic attempt at quantifying the additional cost of ED care during the pandemic for young people with EDs in Canada. This information is vital for providing policy-makers data about ED healthcare spending in the context of high demand for services.

The results from this study highlight a call to action to create mechanisms to learn in real time when more investments in ED services for young people in Canada are needed given shifting needs, as was seen with the pandemic. It additionally suggests that it is vital to close key gaps in the availability of surveillance data to accurately and comprehensively measure the costs associated with ED care. There is an opportunity for ED experts and decision-makers to prioritize working together to create a national surveillance strategy. Using surveillance data will be key to inform timely public health actions, preparedness, and resource allocation for young people with ED and their families in non-crisis and future public health crisis times.

### Electronic supplementary material

Below is the link to the electronic supplementary material.

Supplementary Material 1

## Data Availability

The authors confirm that the data supporting the findings of this study are available within the article and/or its supplementary materials.

## Abbreviations

CIHI: Canadian Institute Health Information
ED: Eating Disorder
EDNS: Eating Disorders Nova Scotia
EDSNA: Eating Disorder Support Network of Alberta
FY: Fiscal Year
NEDIC: National Eating Disorder Information Centre
NIED: National Initiative for Eating Disorders
