# Supplementary material for: Estimating additional health and social costs in eating disorder care for young people during the COVID-19 pandemic: implications for surveillance and system transformation
Source: J Eat Disord. 2024 Apr 26;12:52. doi: 10.1186/s40337-024-01003-1 (PMC11047001; doi:10.1186/s40337-024-01003-1)
Supplement: Supplementary file 1 — Supplementary Material 1 [file 40337_2024_1003_MOESM1_ESM.docx]

Table 1. Summary of eating disorder (ED) cost components used by Deloitte Access Economics to estimate the changes in health system service need, care delivery pathways and economic cost impact across the COVID-19 pandemic.

| Cost component | Source of data | Method of estimation | Cost Reference | Findings | Limitations | |
| --- | --- | --- | --- | --- | --- | --- |
| Change in need for health system services: Comparison of services made between FY20 to FY22 using inpatient admission data, hospital outpatient appointments, and emergency department visits. | | | | | | |
| Inpatient admissions and average length of stay for inpatient admissions | Inpatient admissions and length of stay from CIHI data across FY20-FY22 available for all provinces and territories. | Total number of hospital visits attributed to EDs were multiplied by average costs per visit. Unit costs used in analysis were adjusted for inflation based on changes to health care prices over time using Statistics Canada data. | The unit cost of inpatient hospital visits was based on CIHI’s patient cost estimator ($2021) of $18,165-$29,277 per standard hospital stay for people with EDs. | The number of inpatient hospitalizations with a main diagnosis of an ED was 44% higher in FY21 (2187 visits) and 75% higher in FY22 (2644 visits) than in FY20 (1514 visits). Length of stay for inpatient hospitalizations in AN declined from an average of 25 days (FY20) to 19 days (FY21 and FY22). | Due to data limitations, only length of stay for people with Anorexia Nervosa were included in analysis. Unit costs per standard hospital stay for people with EDs are not specific to children and young people. 48% of inpatient data was suppressed. | |
| Emergency department presentations | Emergency department visits from CIHI data across FY20-FY2022 for available provinces. | Total number of emergency department presentations attributed to EDs were multiplied by average costs per presentation. Unit costs used in analysis were adjusted for inflation based on changes to health care prices over time using Statistics Canada data. | The unit cost of emergency department visits was estimated at ($2021) of $315 per presentation based on CIHI data. | The number of emergency department presentations pre-COVID-19 pandemic were 1019 (FY20). During the COVID-19 pandemic emergency department presentations were an annual average of 2301 (average FY21 and FY22). | Data only available from Ontario, Quebec, and Alberta (extrapolation to other provinces and territories required). Unit costs per emergency department presentation for people with EDs are not specific to children and young people. 46% of data suppressed. | |
| Outpatient appointment utilization | Outpatient appointments from CIHI data across FY20-FY2022 in Alberta. Virtual care visits from the National Physician database. | Total number of outpatient appointments attributed to EDs were multiplied by average costs per appointment. Virtual care visits were estimated from the National Physician database to estimate comparative changes to virtual care use for the period before COVID-19 and the period during the pandemic. Proportions of virtual visits were multiplied by CIHI data on in-person outpatient visits to estimate number of outpatient services. Unit costs used in analysis were adjusted for inflation based on changes to health care prices over time using Statistics Canada data. | The unit cost of outpatient appointments was estimated ($2021) at $115 per appointment based on the National Physician database | 148,252 outpatient appointments conducted in FY20 with only 481 conducted virtually (0.3%). In FY21, 83,167 outpatient visits were completed in person and 51,963 were completed virtually (a 10,702% increase) and in FY22 58,974 visits were completed virtually (a 12,159% increase) | Data available from Alberta only (extrapolation for other provinces and territories required). Dataset is not condition-specific, the approach assumed that the transition to virtual appointments for children and youth with EDs was comparable to those experiencing other conditions. Assumption that all outpatient visits are ED specific. Additionally, only appointments with a physician were included as other data was not available. Therefore, appointments with mental health specialists, support workers, dietitians or other ED specialists not included | |
| Change in care delivery pathways: Use of the average length of stay, shift to virtual care, and wait time data from FY20 to FY22 to understand change in care delivery pathways. | | | | | |  |
| Wait times | Data collected from the Fraser Institute on average wait times across FY20-FY22. | Data collected from the Fraser Institute to estimate average delay accessing specialist ED programs. Average wait time was compared to the median reasonable wait time for access. The total backlog of patients waiting for treatment was estimated based on average annual inpatient admissions from CIHI data. |  | The average wait time in 2019 was approximately 19 weeks to see a specialist in an ED program. In 2022, average wait time was approximately 23 weeks. In 2022, wait times varied from 9 weeks in Saskatchewan to 39 weeks in Manitoba. | CIHI data on wait times for treatment were based on publicly available survey data from the Fraser Institute. Low response rate in Fraser Institute data from Canadian physicians (7.1% response rate in 2022) likely underestimating estimates. The data was not granular enough to examine specific lockdown/restriction impacts. | |
| Economic cost impact: Use of multiple applicable unit costs (see above) to determine changes to health system costs and wait times multiplied by daily cost of living with ED to determine loss of wellbeing. | | | | | | |
| Loss of wellbeing | Loss of wellbeing was based on the cost of additional time an individual spends with an untreated ED. | To estimate the total cost of increased waiting times, the difference between the median wait time to access an ED program and the estimated reasonable wait time was calculated to produce the additional days each person lives with an untreated ED. The total number of days lived with untreated EDs was then multiplied by the estimated daily wellbeing cost of an ED. This is the value of statistical life year (VSLY) multiplied by the relevant disability weight and divided by 365 to adjust to a daily rate. | Using the World Health Organization’s burden of disease methodology, Disability adjusted life years (DALYs) are calculated by assigning disability weights to various states of health. Disability weights for EDs were obtained from IMHE Global burden of disease (AN, BN, and Other or Unspecified Feeding and Eating Disorders). DALYs converted into CDN dollars is based on the VSLY. Estimates inflated to 2023 terms were determined to be $476,000 CAD. | The total cost of wait times on patient outcomes was estimated to be $40.8 million in FY20 and increasing to $57.5 million in FY22. | Considers only wellbeing costs and does not consider additional economic costs that incur such as readmissions, productivity losses, etc. | |

*Reproduced with permission from Deloitte Access Economics; further details available at XX weblink
